# A 30-Year Single-Centre Series of Unknown Primary Merkel Cell Carcinoma: Management and Prognosis

**DOI:** 10.3390/cancers18152368

**Published:** 2026-07-23

**Authors:** Aikaterini Bini, Roxana Totorean, Hemant Kumar, Titus Grecu, Patrick Shenjere, Deemesh Oudit

**Affiliations:** 1 Department of Plastic Surgery, The Christie NHS Foundation Trust, Manchester M20 4BX, UK; aikaterini.bini@nhs.net (A.B.); roxana.totorean@nhs.net (R.T.); h.kumar1@nhs.net (H.K.); titus.grecu@nhs.net (T.G.); 2 Department of Pathology, The Christie NHS Foundation Trust, Manchester M20 4BX, UK; p.shenjere@nhs.net

**Keywords:** Merkel cell carcinoma, unknown primary, lymphatic metastasis, nodal metastasis, prognosis, survival

## Abstract

Background: Merkel cell carcinoma (MCC) is a rare, aggressive cutaneous neuroendocrine malignancy. Like cutaneous malignant melanomas, a subset of MCCs can clinically present without an identifiable primary tumour, termed Merkel cell carcinoma of unknown primary (UPMCC), with incompletely understood behaviour and prognosis. Our objectives are to evaluate the clinical presentation, management and survival outcomes in UPMCC and compare overall survival with metastatic MCC of known-primary origin. Methods: A retrospective review of 252 consecutive MCC patients (1992–2023) identified 20 cases of histologically confirmed nodal or metastatic UPMCC. Demographics, anatomical distribution, treatment and oncological outcomes were analysed. Overall survival was compared with patients presenting with metastatic MCC of known primary. Results: The cohort included 15 males and 5 females (mean age 76 years). Presentation most commonly involved inguinal (*n* = 7) and axillary (*n* = 6) nodes, followed by parotid and cervical basins (*n* = 4). Management was multimodal, including lymphadenectomy, radiotherapy and systemic therapy. Six patients remained disease-free at a mean follow-up of 63.3 months. The mean overall survival was 43.65 months for UPMCC versus 39.29 months for known-primary metastatic MCC. Conclusions: UPMCC most commonly presents as inguinal or axillary nodal disease. Survival outcomes suggest a trend toward improved prognosis compared to known-primary metastatic MCC.

## Simple Summary

Unknown-primary Merkel cell carcinoma (UPMCC) is a rare presentation in which metastatic MCC is detected in lymph nodes without an identifiable skin tumour. Due to its rarity, the clinical behaviour and outcomes of UPMCC remain poorly understood. Previous studies suggest patients with UPMCC may have similar or even better survival than those with known-primary MCC at the same stage. This single-centre UK study adds long-term outcome data after reviewing 252 patients treated for MCC at a single tertiary cancer centre between 1992 and 2023. Twenty patients with UPMCC (8%) were identified. Clinical presentation, treatment strategies and survival outcomes were analysed. Patterns of nodal involvement and multimodal management were detailed. UPMCC demonstrated slightly improved overall survival compared with metastatic MCC of known primary. The findings support a potential survival advantage and reinforce the role of specialist multidisciplinary approach.

## 1. Introduction

Merkel cell carcinoma (MCC) is an uncommon, aggressive neuroendocrine tumour of the skin that most often affects elderly, fair-skinned adults. It is strongly associated with ultraviolet exposure and, in many cases, integration of Merkel cell polyomavirus (MCPyV) into the tumour genome [1]. Merkel cell carcinoma of unknown-primary origin (UPMCC) denotes the clinical scenario in which metastatic MCC is identified, most commonly in regional lymph nodes, without any detectable cutaneous primary tumour on clinical examination or imaging [2]. The global incidence of UPMCC has not been established, as cancer registries record Merkel cell carcinoma without routinely differentiating between known- and unknown-primary presentations. Nevertheless, UPMCC is estimated to represent approximately 5–15% of all MCC cases reported in large institutional series [2]. Although the reason for this behaviour is unknown, it also occurs in other aggressive types of skin cancer such as malignant melanoma [3]. Despite the classic presentation of MCC as a rapidly growing, painless, red-to-violaceous nodule on sun-exposed skin, UPMCC typically appears as an isolated nodal mass (commonly in head and neck, axillary or inguinal lymph nodes) and therefore can be mistaken for other small-cell or neuroendocrine nodal metastases, until immunohistochemistry and molecular testing confirm the diagnosis [4]. Histopathologic diagnosis usually relies on neuroendocrine markers (synaptophysin, chromogranin A, CD56), CK20 positivity with a characteristic perinuclear dot-like pattern and negative TTF-1 to help distinguish UPMCC from metastatic small-cell lung carcinoma. These immunohistochemistry panels are specifically recommended in contemporary practice guidelines [5].

The biological explanation for the absence of a detectable primary lesion in UPMCC is debated; proposed mechanisms include complete spontaneous regression of a small cutaneous primary (potentially immune-mediated), the presence of an occult primary below the resolution of examination or imaging, or the possibility of primary nodal transformation from ectopic Merkel cell precursors or dermal progenitors [6,7]. The same hypotheses have been reported for melanomas with unknown-primary origin. Unknown-primary malignant melanoma (UPMM) is most commonly believed to arise from a regressed primary cutaneous melanoma. There is a less popular theory supporting that UPMM arises from naevi cells in the lymph nodes. The fact that the phenomenon of unknown-primary origin nodal metastasis also occurs in MCC strengthens the argument for a regressed primary tumour, as MCCs, like cutaneous melanomas, are known to regress and also, no established population of Merkel cell precursors is known to reside in lymph nodes [3].

Intriguingly, several retrospective series and multi-institutional analyses suggest that patients presenting with nodal MCC with unknown skin primary often have equal or paradoxically better survival than those with a known cutaneous primary at the same stage. This finding is hypothesised to reflect a more active host antitumour immune response in UPMCC which contributes to spontaneous regression of the primary MCC [8]. However, outcomes vary between cohorts and optimal management strategies remain an area of active research [9,10].

The multimodal management of UPMCC typically follows the principles applied to nodal or regional MCC. Sequentially, this includes a thorough skin examination with dermatologic imaging, ultrasound-guided FNA biopsy for pathological confirmation with appropriate immunohistochemistry, consideration of therapeutic regional lymph node dissection and adjuvant radiation therapy to involved nodal basins. In advanced or unresectable disease, systemic therapy, including immune checkpoint inhibitors, has become a backbone of treatment [5,11].

The purpose of this study is to retrospectively review the patterns of behaviour and the UPMCC-specific overall survival compared to metastatic Merkel cell carcinoma of known primary, in one of the largest single-centre case series of UPMCC.

## 2. Materials and Methods

The selection criteria included all patients presenting to our large tertiary referral centre between 1992 and 2023, with confirmed nodal or distant metastases of MCC without an identified cutaneous counterpart. Data collected included anatomical areas involved, histopathology reports, imaging investigations, multidisciplinary team discussions and management strategies including surgery, radiotherapy or systematic treatment. This informed our analysis to provide a comprehensive understanding of the behaviour of UPMCC.

The disease-specific survival of UPMCC was compared to that of metastatic MCC of known-primary origin (KPMCC) using patients from the same database and timespan. The disease-specific survival was calculated from the date of diagnosis to the date of death attributable to Merkel cell carcinoma. Patients who were alive at the last review or who died from causes unrelated to Merkel cell carcinoma were censored at the date of their last clinical assessment or death. The date of diagnosis was defined as the date of onset of clinically palpable lymphadenopathy; information provided by the patients or the referring hospitals. A Kaplan–Meier survival curve was used to demonstrate the difference in survival rates between UPMCC and KPMCC.

The study interval covers a period of 30 years (1992–2023). During this period major changes in imaging, pathology and immunotherapy have occurred and patients diagnosed with UPMCC 30 years ago may have been treated differently than nowadays, which may have a different impact on the survival rates. The overall changes in therapeutic approach for UPMCC over the last decades are acknowledged as a major limitation of this study.

## 3. Results

During the study interval (1992–2023) the medical records of 252 consecutive patients with MCC were reviewed. Amongst them, 20 cases with UPMCC (7.94%) and 107 cases of metastatic MCC of known primary (42.46%) were recorded. All cases were evaluated and treated within the established multi-disciplinary team approach. There were 15 male (75%) and five female patients (25%) with UPMCC with an average age of 76 years (age range from 66 to 88 years old) upon diagnosis. A summary of the demographics and disease characteristics in this study are provided in Table 1. Table 2 summarises individual patient presentations.

**Figure 1:**
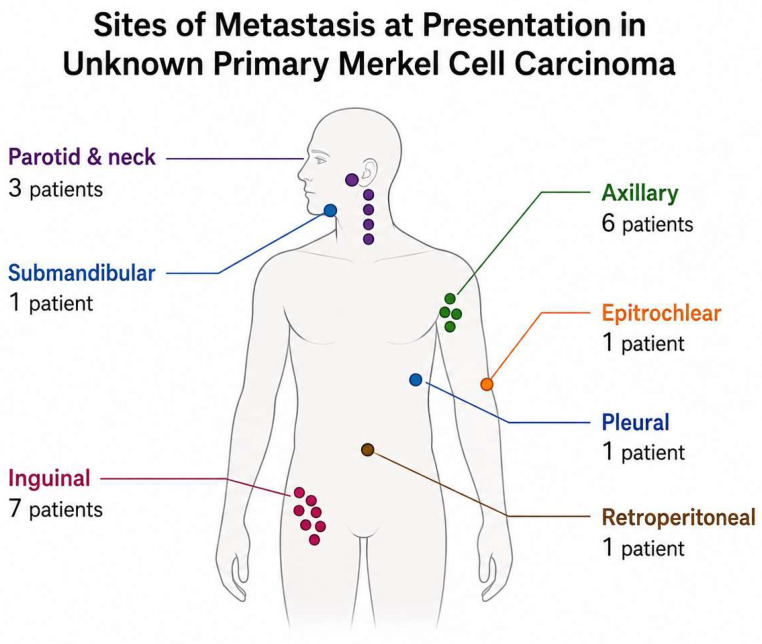
Anatomical sites of initial nodal metastasis of UPMCC.

The anatomical site of the initial nodal metastasis varied. Seven patients (35%) presented with inguinal lymph nodes and we also report cases involving the pelvis, buttock and testicle. Intriguingly, in one observed case of inguinal lymphadenopathy, a metastatic deposit was found in the eyelid at the patient’s first presentation. Nodal disease in the axilla was identified in six patients (30%). There were three patients (15%) with parotid and neck lymphadenopathy and one case with submandibular metastatic nodal MCC (5%). Other uncommon anatomical areas of UPMCC involved the epitrochlear (*n* = 1), retroperitoneal (*n* = 1) and pleural (*n* = 1) lymph nodes (Figure 1).

The multidisciplinary management of patients depended on the stage of the disease (Stage III versus Stage IV), the resectability of the tumour and the performance status of the patient.

Inguinal lymph node dissection was performed in three cases, combined with orchidectomy in a patient with right testicle involvement. Four patients underwent axillary dissection, while parotidectomy and modified radical neck dissection was performed in one patient.

Inoperable inguinal and/or retroperitoneal lymphadenopathy (*n* = 2) and retroperitoneal metastatic disease from head and neck UPMCC (*n* = 1) were treated with radiotherapy. Unresectable axillary lymphadenopathy (*n* = 1), extended parotid and neck UPMCC (*n* = 1) and Stage IV axillary UPMCC with extended disease including supraclavicular, thoracic, mediastinal, para-aortic, inguinal, liver and bone metastases (*n* = 1) were also treated with radiotherapy.

Immune checkpoint inhibitors used included avelumab, nivolumab and atezolizumab as single (*n* = 5) or combined immunotherapy (*n* = 2). Carboplatin and etoposide were the most common chemotherapeutic agents used (*n* = 6). A combination of vincristine sulfate, dactinomycin (Actinomycin-D) and cyclophosphamide (VAC chemotherapy) was used in one case with extended lymphatic, visceral and bone metastatic disease.

Combined treatment modalities were also selected. Therapeutic lymphadenectomy and adjuvant radiotherapy was performed in five patients, followed by adjuvant immunotherapy in three of these cases. Radiotherapy combined with systemic treatment (immunotherapy and/or chemotherapy) was administered in four patients with distant metastases (Stage IV) and four patients with locally advanced and/or inoperable disease (Stage III). A combination of immunotherapy and chemotherapy was given in three cases.

There were also two patients where no treatment was offered due to the extent of the disease, the poor prognosis and their frailty, who died within a month from diagnosis.

All patients had regular follow-up with clinical examination and imaging surveillance, where disease-free status was defined as no clinical and no radiological detectable disease. Currently, six patients are still alive and disease-free with mean time from diagnosis to present approximately 63.33 months. Nine patients died due to progression of the locally advanced disease and/or distal metastases, whilst in five cases death was unrelated to UPMCC.

**Figure 2:**
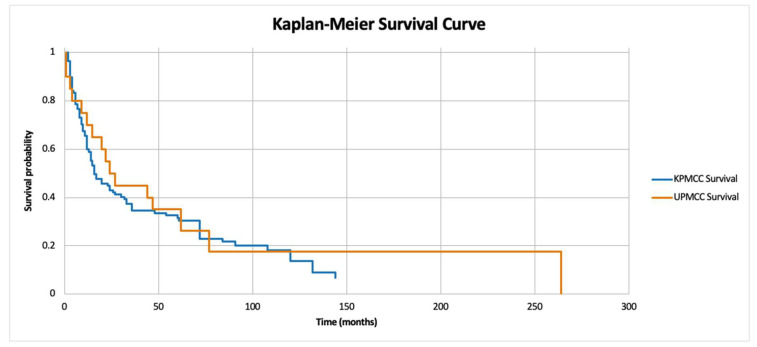
Kaplan–Meier survival curve for UPMCC and KPMCC.

Of 127 patients included in the analysis, 104 (81.9%) died during follow-up and 23 (18.1%) were censored. Kaplan–Meier analysis yielded a median survival of 16.0 months (95% CI: 12.0–30.0) in KPMCC (*n* = 107) and 27.0 months (95% CI: 9.0–62.0) in UPMCC (*n* = 20). As seen on the graph (Figure 2), UPMCC and KPMCC showed similar survival in the first three months, while UPMCC showed higher survival probability through the middle follow-up period (6–90 months). The log-rank test showed no statistically significant difference in survival between groups (χ^2^ = 0.10, df = 1, *p* = 0.75); the wide confidence interval around the UPMCC median survival reflects its small sample size (*n* = 20).

Descriptive comparison tables of UPMCC and KPMCC with Stage III and Stage IV as final disease are provided below (Table 3 and Table 4). 

## 4. Discussion

This study characterised the behaviour of UPMCC, the anatomical areas involved and the disease-specific survival as observed in our large tertiary cancer centre. The different treatment options as well as the prognosis of the disease were carefully analysed in order to provide a comprehensive understanding of the optimal management strategies.

Merkel cell carcinoma of unknown-primary origin most commonly presents as isolated nodal disease, with the inguinal/iliac basins reported in the majority of series followed by axillary, epitrochlear, parotid and cervical nodal basins. This is contrary to Merkel cell carcinoma of known-primary origin that occurs most frequently in the head and neck region [12,13]. Pathology reports and case series emphasise that MCC tissue found in a lymph node is morphologically and immunophenotypically indistinguishable from cutaneous MCC, leaving it unclear whether some nodal cases represent occult/regressed skin primaries or very rare primary nodal transformation [14]. Imaging and careful dermatologic and mucosal examination focus on the commonly involved anatomical regions (head and neck, upper limbs/axilla, lower limbs/groin) and include cross-sectional imaging or PET-CT aiming at identification of occult primaries or distant disease [7].

Surgical management of UPMCC follows the same regional principles as for nodal or locoregional MCC. When a nodal mass is resectable, complete therapeutic lymphadenectomy is the cornerstone of management [15,16]. Adjuvant radiotherapy to the involved nodal basin is frequently recommended after surgery, especially in head and neck disease or when close margins or extracapsular extension are present. Retrospective series have shown improved regional control with postoperative radiotherapy [16].

In unresectable, recurrent or metastatic UPMCC to distant areas the treatment emphasis shifts to systemic therapy. Immune checkpoint inhibitors are now the preferred first-line option for most patients, as they produce higher and more durable response rates than classic chemotherapy. Avelumab (anti-PD-L1) demonstrated clinically meaningful objective response rates and durability in pivotal trials and is approved for metastatic MCC, while pembrolizumab (anti-PD-1) is also supported by guideline inclusion and trial evidence [17,18]. Conventional cytotoxic chemotherapy (typically platinum-based regimens such as carboplatin or cisplatin combined with etoposide) can induce rapid responses and is still used for palliation or where immunotherapy is contraindicated, but responses are often short-lived and associated with greater toxicity than immune checkpoint inhibitors [19]. Radiotherapy remains an important non-surgical tool both for definitive treatment of unresectable nodal disease as well as for palliation. It is commonly integrated with surgery and systemic therapy to maximise local control in multidisciplinary plans tailored to the patient’s stage, performance status and immune competence [20].

Patients with UPMCC, both metastatic or nodal MCC without an identifiable cutaneous primary, appear to have a significantly better prognosis than patients with a known cutaneous primary MCC at a similar stage. There is possibly an unknown pathway at a cellular and molecular level that may explain the difference in prognosis due to the difference in the tumour microenvironment (TME) of both groups of tumours, e.g., primary MCC and UPMCC [6,21,22,23]. In a retrospective series of Stage III disease, patients with nodal UPMCC had a median overall survival “not reached” compared with a median overall survival of 21 months in those with a known-primary MCC (hazard ratio = 0.34; *p* = 0.03), indicating a roughly two-thirds reduction in risk of death [9]. In that same study, disease-free survival (recurrence-free) was also improved: the median disease-free survival was not reached in the UPMCC group versus 15 months in the known-primary MCC group (hazard ratio = 0.48; *p* = 0.18 for recurrence-free survival).

More recently, a larger pooled analysis of 123 patients with advanced MCC (Stage IIIb or IV) reported that UPMCC patients were nearly twice as likely to survive compared with similarly staged known-primary MCC patients. In multivariate analysis, UPMCC status was associated with a substantially reduced risk of MCC-specific death (hazard ratio 0.297; *p* < 0.001) [24]. Even among patients presenting with distant metastatic disease (Stage IV), those with UPMCC had better MCC-specific survival than their known-primary counterparts (hazard ratio = 0.296; *p* = 0.038) [24]. A more recent single-centre cohort including 55 patients with exclusive nodal UPMCC and no distant metastases found a 5-year cancer-specific survival (CSS) rate of 68.5% (95% CI: 52.8–79.9%) and a 5-year relapse-free survival (RFS) of 56.6% (95% CI: 42.0–68.8%) [7].

Biologically, these survival differences may reflect distinct tumour and host-immune characteristics in UPMCC. Tumours from UPMCC patients, either virus (MCPyV)-positive or -negative, often show similarly high immunogenicity compared to known-primary MCC, including comparable or even higher levels of intratumoral CD8+ T-cells and PD-L1 expression [6]. Moreover, in virus-negative UPMCC, next-generation sequencing reveals UV-signature mutations and high tumour mutational burdens, akin to what is seen in cutaneous MCC, which may also contribute to increased immunogenicity and better immune-mediated tumour control [6,24]. One leading hypothesis is that a robust host immune response may lead to spontaneous regression of the primary skin tumour, thereby leaving only the metastatic node, and that this same immunity continues to control residual disease, improving survival [24,25]. Differences in the cellular and molecular aspects of the TMEs may also be considered to support the favourable survival in UPMCC [6,21,22,23].

Studies of Stage IIIb nodal Merkel cell carcinoma consistently show that patients presenting with an occult primary have significantly better survival than those with a known primary, indicating that the absence of a detectable skin lesion confers a favourable prognostic implication in nodal disease [3,26,27]. Retrospective cohort analyses of lymph node positive cases demonstrate that patients with an unknown primary can achieve strong disease control and favourable survival when managed with appropriate nodal surgery and adjuvant radiotherapy, reinforcing the pattern of improved prognosis for this subgroup [28]. Population-level data from the immunotherapy era also reveal differing survival trends between known- and unknown-primary Merkel cell carcinoma, examining whether immune checkpoint inhibitors have altered the long-observed survival advantage associated with unknown-primary disease [29].

However, “better prognosis” does not mean “good prognosis.” In a large series of 415 patients with MCC, the subgroup with UPMCC (about 9% of cases) still showed a substantial risk of recurrence and death. Many of those patients presented with nodal disease (or parotid gland involvement) and 24.3% had distant metastases at diagnosis, while the 5-year disease-specific survival in that series was 63.3% [11]. Thus, despite their relative advantage over known-primary disease, patients with UPMCC still face significant long-term risks, underscoring the need for careful long-term follow-up and aggressive management within the multidisciplinary team of tertiary referral centres.

Although our findings support the growing body of evidence that UPMCC is associated with more favourable survival than stage-matched MCC with a known primary, the biological mechanisms responsible for this observation remain incompletely understood [6,24]. Current evidence suggests that the survival advantage is unlikely to be explained solely by differences in treatment strategy and instead reflects important interactions between tumour biology and host immunity [6,21,23,24]. Comprehensive molecular analyses have demonstrated that UPMCC includes both MCPyV-positive tumours characterised by abundant immune-cell infiltration and MCPyV-negative tumours exhibiting ultraviolet-signature mutations and high tumour mutational burden, both of which may enhance tumour immunogenicity [6,24]. These observations support the hypothesis that an effective immune response may eradicate the primary cutaneous lesion while simultaneously suppressing metastatic progression, thereby contributing to the improved outcomes consistently reported in UPMCC [6,8,24]. Nevertheless, the precise cellular interactions within the tumour microenvironment, including the roles of cytotoxic T lymphocytes, regulatory T cells, tumour-associated macrophages and immune checkpoint pathways, require further investigation before reliable prognostic biomarkers can be incorporated into routine clinical practice [21,22,23,30].

Another important area of future research is the development of personalised surveillance strategies using molecular biomarkers [20,30]. Recent studies have demonstrated that circulating tumour DNA (ctDNA) has considerable potential as a highly sensitive biomarker for detecting minimal residual disease, monitoring treatment response and identifying recurrence earlier than conventional imaging in patients with Merkel cell carcinoma [30,31]. When integrated with clinicopathological variables, radiological assessment and established prognostic factors, ctDNA may facilitate more individualised follow-up protocols and allow earlier therapeutic intervention following disease relapse [30,31]. Similarly, further characterisation of MCPyV status, tumour mutational burden, PD-L1 expression and the composition of the immune microenvironment may improve risk stratification and help identify patients most likely to benefit from immune checkpoint inhibition or combined treatment approaches [6,21,22,24,30]. As immunotherapy continues to evolve, incorporation of molecular biomarkers into prospective clinical studies may further refine treatment selection and improve long-term oncological outcomes for patients with both known-primary and unknown-primary MCC [18,20,30].

Given the rarity of UPMCC, meaningful advances in clinical management will almost certainly require international collaboration and prospective multicentre data collection [7,20]. Most published evidence continues to originate from retrospective single-institution series with relatively limited patient numbers, restricting statistical power and introducing unavoidable selection bias [7,9,11,27,28]. Establishment of international registries with standardised reporting of clinicopathological variables, treatment protocols, molecular characteristics and long-term oncological outcomes would substantially strengthen the evidence base and facilitate more robust comparisons between treatment strategies [20,31]. Such collaborative efforts may also clarify the optimal sequencing of surgery, radiotherapy and systemic immunotherapy, particularly for patients presenting with advanced nodal disease or synchronous distant metastases [18,20]. Until higher-level evidence becomes available, management should continue to be individualised within experienced multidisciplinary teams capable of integrating surgery, radiation oncology, medical oncology, pathology, dermatology and specialised imaging to optimise patient outcomes [5,20].

The present study has limitations. Although the extended duration of data collection, spanning more than 30 years, is a key strength, this also introduces inherent challenges. Over this period, clinical practices have evolved substantially, including advances in diagnostic modalities, treatment strategies and surgical techniques. These temporal changes introduce potential confounding factors that may influence the interpretation of outcomes. Additionally, the rarity of the condition limits the ability to achieve a large sample size. This constraint is further compounded by the focus on a specific subset of the cohort, namely patients with unknown-primary Merkel cell carcinoma, which reduces the overall number of cases available for statistical analysis.

## 5. Conclusions

Merkel cell carcinoma of unknown-primary origin appears to carry a better prognosis than metastatic MCC with a known-primary origin for both regionally metastatic (nodal) disease and distant metastatic disease. This advantage is likely mediated, at least in part, by more active host immune responses and higher immunogenicity, potentially resulting in elimination or regression of the primary tumour and better control of metastatic disease. Still, UPMCC remains a serious, potentially fatal disease, with appreciable recurrence and long-term risk. Therefore, clinical vigilance and multidisciplinary approach remain essential.

## Figures and Tables

**Table 1:** Patient demographics and disease characteristics (UPMCC cohort).

Variable	Value
Number of patients	20
Study period	1992–2023
Mean age at diagnosis (range)	76 years (66–88)
Sex	Male: 15 (75%) Female: 5 (25%)
Initial site of nodal involvement	Inguinal: 7 Axillary: 6 Parotid and neck: 3 Submandibular: 1 Epitrochlear: 1 Retroperitoneal: 1 Pleural: 1
Disease stage at presentation	Stage III (nodal): 14 Stage IV (distant metastases): 6
Follow-up duration (mean)	63.3 months
Patients alive at last follow-up	6 (30%)
Cause of death	UPMCC-related: 9 Unrelated to UPMCC: 5

**Table 2:** Patient demographics, anatomical area of UPMCC, surgical and non-surgical management and disease-specific survival.

Gender	Age	Anatomical Area	Surgery	Radiotherapy	Immunotherapy	Chemotherapy	Survival
Male	71	Left groin	Left groin dissection	Adjuvant	-	-	Still alive 41 months DF
Male	66	Right axilla	Right axillary dissection	Adjuvant	-	-	Still alive 49 months DF
Male	77	Right submandibular lymph nodes	No Liver metastasis	-	Avelumab and Atezolizumab	Carboplatin and Etoposide	Still alive 55 months DF
Male	79	Right buttock	No Pelvic lymphadenopathy	-	Avelumab	-	Death in 4 months, not UPMCC-related
Female	66	Left elbow/epitrochlear lymph nodes	Wide local excision	-	-	-	Still alive 62 months DF
Female	79	Right parotid and neck	MRND and parotidectomy	Adjuvant	Avelumab	-	Death in 44 months, retroperitoneal metastasis, UPMCC-related
Male	75	Left groin and pelvis	No Inoperable	Neo-adjuvant	Avelumab	Carboplatin and Etoposide	Death in 47 months, UPMCC-related
Male	69	Right axilla and right upper arm metastatic deposit	Right axillary dissection and resection of right upper arm deposit	Adjuvant	Avelumab	-	Death in 27 months, left adrenal metastasis, UPMCC-related
Male	69	Right axilla	Right axillary dissection	Adjuvant	Avelumab	-	Still alive 77 months DF
Male	70	Retroperitoneal	No Inoperable	-	-	-	Death in 1 month, UPMCC-related
Male	77	Inguinal lymph nodes and eyelid metastatic deposit	-	-	-	Carboplatin and Etoposide	Death in 3 months, UPMCC-related
Female	83	Right groin	Right groin dissection	-	-	-	Death in 24 months, not UPMCC-related
Male	83	Right groin	No Inoperable	Neo-adjuvant	Avelumab and Nivolumab	-	Death in 9 months, UPMCC-related
Female	74	Left axilla	-	Neo-adjuvant	Avelumab	Carboplatin and Etoposide	Still alive 96 months DF
Male	83	Left parotid and neck	No Abdomen and brain metastases	Radiotherapy and SRS for brain metastasis	-	Carboplatin	Death in 20 months, UPMCC-related
Male	80	Right parotid and neck	-	Neo-adjuvant	-	-	Death in 22 months, not UPMCC-related
Male	80	Pleural	-	-	-	-	Death in 1 month from metastatic prostate cancer
Female	75	Right axilla	Right axillary dissection	-	-	-	Death in 12 months, mediastinal lymph nodes and adrenal mass, UPMCC-related
Male	75	Right axilla	No Disease widespread	Neo-adjuvant	-	Carboplatin and Etoposide, VAC then Carboplatin	Death in 15 months, supraclavicular, thoracic, mediastinal, para-aortic, inguinal, liver, bone metastases, UPMCC-related
Male	88	Right groin and right testicle	Right groin dissection and right orchidectomy	-	-	-	Death in 264 months (22 years) Not UPMCC related

Abbreviations: DF: disease free, MRND: modified radical neck dissection, SRS: stereotactic radiosurgery, VAC: vincristine sulfate, dactinomycin (Actinomycin-D) and cyclophosphamide.

**Table 3:** UPMCC vs. KPMCC: Descriptive Comparison by Final Disease Stage—AJCC Stage III Disease.

Characteristic	UPMCC	KPMCC
***n*** ** (patients)**	**9**	**61**
Alive	2 (22.2%)	14 (23.0%)
Deceased	7 (77.8%)	47 (77.0%)
**Age (years)**		
Mean (range)	76.2 (67–84)	82.3 (59–96)
Median (range)	76 (67–84)	83 (59–96)
**Primary nodal site, ** ***n*** ** (%)**		
Head	0 (0.0%)	24 (39.3%)
Neck	1 (11.1%)	5 (8.2%)
Trunk	0 (0.0%)	5 (8.2%)
Upper Limb	4 (44.4%)	11 (18.0%)
Lower Limb	4 (44.4%)	16 (26.2%)
**Treatment received, ** ***n*** ** (%)**		
Surgery	2 (22.2%)	40 (65.6%)
SACT (systemic anticancer therapy)	3 (33.3%)	9 (14.8%)
Radiotherapy—primary site	0 (0.00%)	40 (65.6%)
Radiotherapy—draining basin	6 (66.7%)	34 (55.7%)
**Survival, months (deceased only)**		
Median (range)	24 (9–77)	16 (3–215)

**Table 4:** UPMCC vs. KPMCC: Descriptive Comparison by Final Disease Stage—AJCC Stage IV Disease.

Characteristic	UPMCC	KPMCC
***n*** ** (patients)**	**11**	**46**
Alive	2 (18.2%)	4 (8.7%)
Deceased	9 (81.8%)	42 (91.3%)
**Age (years)**		
Mean (range)	79.0 (71–89)	78.3 (54–94)
Median (range)	79 (71–89)	82 (54–94)
**Primary nodal site, ** ***n*** ** (%)**		
Head	2 (18.2%)	17 (37.0%)
Neck	1 (9.1%)	2 (4.3%)
Trunk	2 (18.2%)	6 (13.0%)
Upper Limb	3 (27.3%)	13 (28.3%)
Lower Limb	3 (27.3%)	8 (17.4%)
**Treatment received, ** ***n*** ** (%)**		
Surgery	1 (9.1%)	22 (47.8%)
SACT (systemic anticancer therapy)	8 (72.7%)	17 (37.0%)
Radiotherapy—primary site	0 (0.0%)	28 (60.9%)
Radiotherapy—draining basin	5 (45.5%)	24 (52.2%)
**Survival, months (deceased only)**		
Median (range)	15 (1–264)	12 (2–144)

## Data Availability

Restrictions apply to the data set: data underlying this article cannot be shared publicly due to it being stored within the hospital database of the Christie NHS Foundation Trust, to maintain confidentiality and anonymity of patients reported on. Data sharing can only be considered upon a reasonable request to the corresponding author.
